# Effects of dietary supplementation with milk fat globule membrane on the physical performance of community-dwelling Japanese adults: a randomised, double-blind, placebo-controlled trial

**DOI:** 10.1017/jns.2018.8

**Published:** 2018-04-19

**Authors:** Yasuo Kokai, Nana Mikami, Mitsuhiro Tada, Kazuichi Tomonobu, Ryuji Ochiai, Noriko Osaki, Yoshihisa Katsuragi, Hitoshi Sohma, Yoichi M. Ito

**Affiliations:** 1 Department of Biomedical Engineering, Sapporo Medical University School of Medicine, S1W17, Chuo-ku, Sapporo, Japan; 2 Department of Neurosurgery, Rumoi Municipal Hospital, 2-16-1 Shinonome, Rumoi, Japan; 3 Health Care Food Research Laboratory, Kao Corporation, Tachibana-2-1-3, Sumida-ku, Tokyo, Japan; 4 Department of Educational Development, Sapporo Medical University Center for Medical Education, S1W17, Chuo-ku, Sapporo, Japan; 5 Department of Biostatics, Hokkaido University Graduate School of Medicine, N15W7, Kita-ku, Sapporo, Japan

**Keywords:** Physical performance, One-leg stand, Balance, Agility, Ageing, Milk fat globule membrane, kwd>ALAT, alanine aminotransferase, kwd>ASAT, aspartate aminotransferase, kwd>MFGM, milk fat globule membrane, kwd>VM, *vastus medialis* of *quadriceps femoris* muscle

## Abstract

We conducted a randomised, double-blind, placebo-controlled trial to elucidate the effects of dietary milk fat globule membrane (MFGM) on the physical performance of community-dwelling Japanese adults. For this 24-week study, 115 middle-aged subjects (range 50–70 years old) were invited, of whom 113 (seventy-two women, forty-one men) completed the trial. Participants were then divided into either the placebo control or MFGM group. Measurements of physical performance (without undertaking any mandatory exercise) examining muscle strength, agility and balance were tested every 6 weeks until 24 weeks. Analyses were performed using the intention-to-treat method for all participants. Although the effects of MFGM on muscle strength and agility were not significant, we noted that the parameter for balance (such as the ability to stand on one leg with eyes closed for longer durations) increased in the MFGM group (mean 10·1 (95 % CI 8·25, 12·4) s) compared with the placebo (mean 7·53 (95 % CI 6·11, 9·30) s) (*P* = 0·046). Similarly, application of the mixed-effect model for repeated measures under unstructured covariance also revealed that the effect of MFGM was significant when compared with the placebo (10·2 (95 % CI 8·33, 12·4) *v.* 7·61 (95 % CI 6·17, 9·30) s) (*P* = 0·045). In conclusion, we demonstrated that MFGM had an effect on the physical performance of community-dwelling Japanese adults despite mandatory exercise. However, studies using larger cohorts of individuals from different demographic backgrounds are required to further elucidate the mechanisms underlying these effects and to extend the application of MFGM.

Longevity has been noted to increase in the elderly population in developed countries^(^^1^^)^. Although ageing negatively affects many aspects of physical performance, the underlaying mechanisms remain unclear^(^^2^^)^. Thus, this limited knowledge has hampered the development of a proper approach to support age-related issues. Physical performance, including muscle strength, balance and agility, are fundamental elements of activity of daily living that play a pivotal role in supporting an active and comfortable lifestyle throughout the stages of life. Ageing deteriorates not only the physical performance, but also an individual's eating ability^(^^3^^)^, and considering the close relationship between these two important functions, it has been suggested that the diet plays an essential role in supporting physical performance especially in ageing individuals.

Ageing is a complex phenomenon affecting various organ systems including the neuromuscular system^(^^4^^–^^6^^)^, where changes in the neuromuscular components of aged animals have been previously shown to negatively affect both the quality and quantity of physical performance^(^^7^^)^. The neuromuscular system is a unique structure containing two independent tissues comprising a skeletal muscle and a peripheral nerve^(^^8^^)^. The synaptic structure of this particular system – termed neuromuscular junctions – plays a critical role in various functions that are essential for active daily living and presents as a possible target for impairment of physical activity by ageing.

Previous studies have assessed the effects of several nutritional components possessing different functions on improving the physical activity of relatively healthy elderly individuals in both mice and man^(^^9^^–^^11^^)^. Recently, our group identified that the administration of milk fat globule membrane (MFGM) mediated a unique and preferable effect on improving the physical performance in mice^(^^10^^–^^13^^)^ and human subjects^(^^14^^–^^17^^)^, which suggests an improvement in muscle mass and strength through the improved functions of neuromuscular junctions in aged individuals with regular exercise. MFGM secreted by the epithelial cells lining the alveolar lumen of the lactating mammary gland contains various enzymes, proteins and lipids, including sphingomyelin^(^^15^^,^^18^^,^^19^^)^. Although these preceding cohort studies strongly suggest an effect of MFGM on physical performance^(^^14^^–^^17^^)^, all participants were required to exercise (intensity of 12–14 on the Borg Rate of Perceived Exertion scale^(^^20^^)^) in addition to their normal physical activity.

In the present study, we aimed to determine the effects of dietary MFGM on the physical performance of community-dwelling Japanese adults. In order to elucidate the effects of only dietary MFGM, all participants were asked to maintain their normal diets and physical activities without any additional exercise throughout the study period.

## Methods

### Materials

MFGM and placebo tablets were prepared by Kao Corporation. The composition for six tablets is shown in Table 1. All tablets were identical in terms of appearance, taste and texture. The major details on MFGM tablets have been described by Ota *et al.*^(^^15^^)^.

**Table 1.** Composition of milk fat globule membrane (MFGM) and placebo in six tablets*

Components	MFGM in six tablets	Placebo in six tablets
Energy (kcal)†	9·3	9·3
Protein (g)	0·53	0·27
Lipid (g)	0·31	0·32
Carbohydrate (g)	1·73	1·99
Na (mg)	1·2	3·2
MFGM (g)	1·0	0
Whole milk powder (g)	0	1·0
Sphingomyelin (mg)	38	1·3

* The study participants were instructed to take six tablets per d for 24 weeks in the morning after their breakfast.

† To convert kcal to kJ, multiply by 4·184.

### Subjects

A total of 115 healthy community-dwelling Japanese adults aged between 50 and 70 years from Rumoi, Hokkaido, Japan were invited for this study. Of this total, 113 participants, including seventy-two women and forty-one men, completed the trial. Written informed consent was obtained from all participants. Those (one man and one woman) unable to give consent were excluded from the study.

### Study design

This randomised, double blind, placebo-controlled, parallel-group clinical trial was conducted at a single centre in Japan from 11 August 2015 to 10 May 2016. Following randomisation procedures (using computerised random numbers^(^^21^^)^), subjects were double-blinded (participants and trial practitioner), and randomly assigned to the two treatment groups (allocation ratio, 1:1) either the placebo or MFGM group. The subjects were instructed to take six tablets once per d for 24 weeks in the morning after their breakfast, and also not to alter their normal diets or physical activities.

This study was conducted according to the guidelines of the Declaration of Helsinki, and our study protocol and all procedures involving human participants were approved by the Sapporo Medical University Research Community (approval number 26-2-58, the protocol was approved on 31 March 2015) and registered on 6 August 2015 with the University Hospital Medical Information Network Clinical Trials Registry (UMINCTR; trial number UMIN000018564, http://www.umin.ac.jp/). This study followed the CONSORT 2010 checklist (http://www.consort-statement.org/).

### Blood biochemical markers

Blood samples were drawn after 12 h of fasting at 0, 12 and 24 weeks of the study. The following levels were measured: leucocytes, erythrocytes, Hb, haematocrit, blood glucose, HbA1c, total cholesterol, LDL-cholesterol, HDL-cholesterol, TAG, aspartate aminotransferase (ASAT) and alanine aminotransferase (ALAT).

By using a vacuum blood collection tube (VenojectII VP-DK052K; Terumo) containing ethylenediamine-N,N,N′,N′-tetraacetic acid, dipotassium salt (EDTA-2K) as anticoagulant, whole blood was collected, and leucocytes, erythrocytes, Hb and haematocrit were analysed with a multichannel blood cell analyser (XN-3000; Sysmex). With a vacuum blood collection tube (VenojectII VP-FH052K; Terumo) containing sodium flouride (NaF) as anticoagulant, either whole blood for HbA1c or plasma for glucose were prepared, and analysed with either an automatic glycohaemoglobin analyser (HLC723G8; Tosoh) or automatic blood glucose analyser (GA08II; A & T Co. Ltd), respectively. Sera were prepared for total cholesterol, LDL, HDL, TAG, ASAT and ALAT with a vacuum blood collection tube without anticoagulant (VenojectII VP-AS076K; Terumo), and analysed by an automatic clinical chemistry analyser (Architect ci16200; Abbott).

Blood glucose is the amount of glucose present in the blood at any given time, whereas HbA1c reflects the cumulative glycaemic history over a period of time (average glycaemia over the previous 2 weeks and 3–4 months, respectively). Total cholesterol, HDL, LDL and TAG reflect lipid metabolism; while HDL, LDL and TAG levels are used for the diagnosis of dyslipidaemia. ASAT and ALAT are enzymes related to liver function and are used to evaluate hepatic toxicity.

### Primary and secondary outcomes

We assessed the isometric knee extension strength of each leg as the indicator of muscle strength, and as the primary outcome of this study. Secondary outcomes were selected to measure some aspects of agility, balance and muscle mass. These included a chair stand test, a chair stepping test, and one-leg standing time with eyes closed measured at 0, 6, 12, 18 and 24 weeks of the study. Muscle mass measured from the *vastus medialis* of *quadriceps femoris* (VM) as an indicator of the muscle cross-sectional area of thigh was calculated using ImageJ distributed by the National Institutes of Health (https://imagej.net/ImageJ) from MRI images taken at 0 and 24 weeks of the study.

The outcome measures, including isometric knee extension strength of each leg, the chair stand test, and areas of VM were measured as follows^(^^15^^)^. In brief, the isometric knee extension strength of each leg was measured as an indicator of a maximal isometric force of the thigh. The participant sat on the bench. The centres of rotation of the tensiometer and knee joint were carefully adjusted. Just before measurement, the participant was asked to extend the knee with maximal effort. Isometric knee extension strength of each leg was measured using a Force Measurement System for One Leg (T.K.K.5715; Takei Scientific Instruments Co.) equipped with a tensinometer D (T.K.K.5710e; Takei Scientific Instruments Co.). The subjects performed two maximal 3-s voluntary contractions at 90° knee flexion with 10 s of rest between each attempt. The average muscle peak strength of each leg was calculated as the maximal isometric knee extension strength. The chair stand test was measured as an indicator of muscular endurance and agility. The participant stood up and sat down five times with their arms folded in front of their chests as quickly as possible on a firm chair. The test was performed with a folding chair without arms. Care was taken to prevent the chair from moving at the test. The participant sat in the middle of the chair and the test was started by ‘start’ as the signal. The time required to complete five cycles was measured twice. The average time was calculated as the chair stand test performance. The areas of VM were measured as an indicator for muscle mass. The MRI of each thigh was obtained using a 1·5 T superconducting system (Achieva 1·5 T Conversion; Philips). The thigh image obtained 100 mm proximal to the knee joint was utilised for calculation of the cross-sectional area using ImageJ (https://imagej.nih.gov/ij/).

The chair stepping test was measured as another indicator of muscular endurance and agility. An armless folding chair was placed directly behind a mat. Participants were asked to sit on the chair, keeping their legs in a parallel position, and rest their feet upon the mat. Their hands were positioned so that their open palms were touching the sides of the chair seat. Participants were then asked to step in place, without allowing their heels to touch the mat, as fast as possible for 10 s. The number of steps was counted using a step counting instrument (T.K.K.5301; Takei Scientific Instruments, Co. Ltd). The test was performed twice, and the participants’ scores were determined by the average of the two tests.

The time for the one-leg stand test with eyes closed was measured as an indicator for balance, and defined as the time measured using a stopwatch from when the subject lifted his or her leg from the floor until they touched the floor or shifted their weight to regain their balance without placing their foot on the floor^(^^22^^,^^23^^)^.

### Statistical analysis

The sample size was calculated from a previous study reporting a significant increase of knee extension as the primary outcome of this trial. This study revealed that the mean difference of changes of knee extension between MFGM and placebo was 2·30 (se 1·31) kg by ANCOVA with sex and baseline measure of knee extension as covariates. For an ANCOVA with sex and baseline measure of knee extension as covariates with two-sided significance level of 0·05, a total sample size of 104 is required to obtain a power of at least 0·8 to detect a mean difference of 2·30. Assuming a 10 % dropout rate, the sample size in this study was set as 115.

Basic characteristics of the study groups were compared using the Student's *t* test, Welch test, Wilcoxon test or χ^2^ test according to the distribution of the variables. The primary and secondary outcomes were assessed using ANOVA at 0 and 24 weeks of the intervention with supplement case as covariate. If significant effects were detected between MFGM and placebo, a mixed model for repeated measures with unstructured covariance was then performed. A significant difference was set at *P* < 0·05. All analyses were performed using JMP^®^ pro13 (SAS Institute Inc.).

## Results

### Characteristics of subjects

**Fig. 1.**
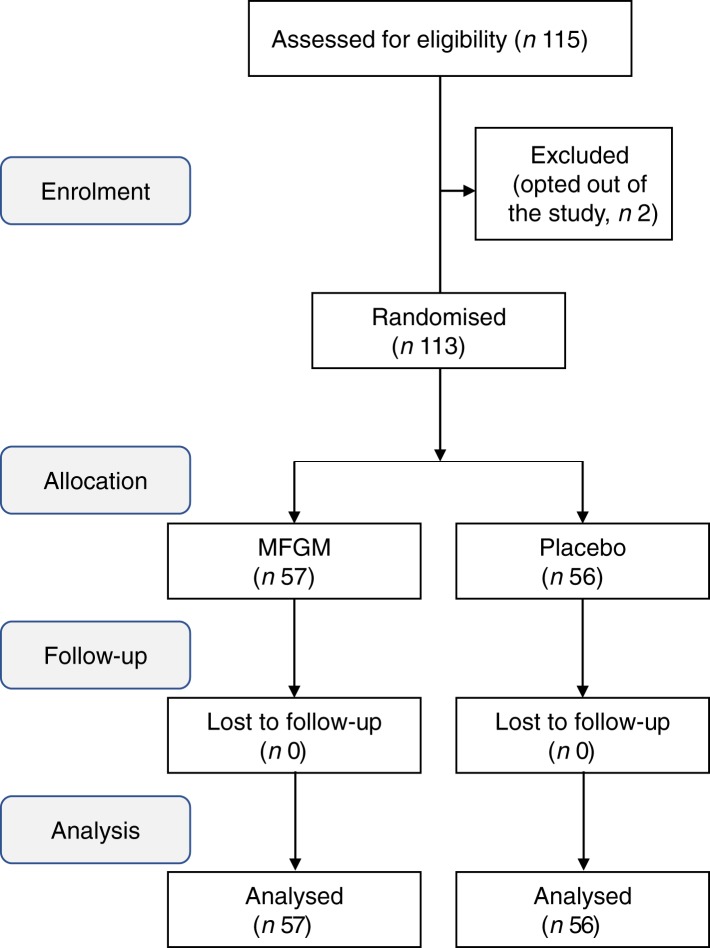
Flow diagram of community-dwelling Japanese adults aged between 50 and 70 years old who were enrolled in the milk fat globule membrane (MFGM) clinical study. Subjects were randomly allocated to the MFGM and placebo groups, and followed up for 24 weeks.

This study was a double-blinded and randomised intervention trial. A total of 113 participants provided informed consent to the aims of this clinical study. The allocation, follow-up and analysis of the study are summarised in Fig. 1.

The subjects were evenly distributed in terms of age, sex, BMI, blood examinations and leg extension at the primary outcome of this study as shown in Table 2. No drop-out was observed. The follow-up rate was 100 % and no adverse events were reported during the study.

**Table 2.** Baseline characteristics of study participants* (Numbers of participants and percentages; mean values and standard deviations)

	MFGM		Placebo		
Variables	Mean	sd	Mean	sd	*P*
Demographic variables					
Subjects					0·96
Number	57	56	
% male	36·2	35·7	
Age (years)	59·6	5·64	60·0	5·61	0·67
Height (cm)	159·0	8·23	159·9	8·16	0·56
Weight (kg)	59·2	12·1	58·9	12·1	0·91
BMI (kg/m^2^)	23·3	3·96	22·9	3·97	0·66
Primary endpoint: isometric knee extension strength of each leg (kg)					
Right	34·3	13·9	33·5	12·4	0·76
Left	33·5	12·4	32·7	12·6	0·71

MFGM, milk fat globule membrane.

* No significant differences were observed between the two groups.

### Milk fat globule membrane did not change the primary outcome of isometric knee extension strength

Dietary MFGM intake did not significantly affect the primary outcome of isometric knee extension strength of each leg. The statistical difference of changes of knee extension between MFGM and placebo was 2·30 kg (*P* = 0·33) (Table 3). In addition to leg extension, measures of chair stand, chair stepping and area of VM did not show any significant changes between the MFGM and placebo groups as shown in Table 3.

**Table 3.** Effects of dietary milk fat globule membrane (MFGM) on physical performance*

	MFGM			Placebo			
Measures	Mean	se	95 % CI	Mean	se	95 % CI	*P*
Isometric knee extension strength of each leg (kg)							
Right	37·3	1·74	33·9, 40·8	34·9	1·78	31·4, 38·4	0·33
Left	35·6	1·75	32·2, 39·1	33·9	1·79	30·4, 37·5	0·50
Chair stand test†	6·65	0·17	6·31, 6·99	6·73	0·17	6·39, 7·07	0·74
Chair stepping test†	90·9	1·75	87·4, 94·4	91·8	1·80	88·2, 95·4	0·72
One-leg stand test with eyes closed‡	10·1	1·11	8·25, 12·4	7·53	1·12	6·11, 9·30	0·046
Area of *vastus medialis* of the *quadriceps femoris*§	26·0	1·01	25·5, 26·6	25·8	1·01	25·3, 26·3	0·28

* Values at 24 weeks of each measure were analysed for statistical differences between the MFGM and placebo groups by ANOVA.

† Number of actions per scheduled time.

‡ Time of one-leg stance with eyes closed (s).

§ Pixels of *vastus medialis* of the *quadriceps femoris* muscle on MRI images.

### Significant increase of one-leg standing time with eyes closed

**Fig. 2.**
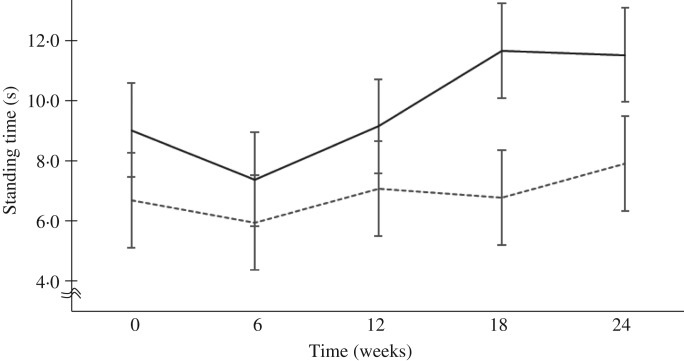
Significant increase in one-leg standing time with eyes closed. One-leg standing time was analysed using a mixed-effect mode with repeated measures. The model includes fixed effects of intervention, time-point, and treatment-time point interaction with repeated covariate structure as unstructured. Values are means, with 95 % confidence intervals represented by vertical bars. In the 24-week trial, one-leg standing time with eyes closed significantly increased in the group supplemented with dietary milk fat globule membrane (MFGM; ––) *v.* placebo (- - -): mean 10·2 (95 % CI 8·33, 12·4) *v*. 7·61 (95 % CI 6·17, 9·30) s (*P* = 0·045).

Although MFGM intake did not significantly change isometric knee extension strength, MFGM supplementation significantly increased the one-leg standing time with eyes closed at 24 weeks of the study (MFGM *v.* placebo; mean 10·1 (95% CI 8·25, 12·4) *v.* 7·53 (95 % CI 6·11, 9·30) s; *P* = 0·046) (Table 3). The mixed-effect model with unstructured covariance was used to further study the effect of MFGM over the study period (0, 6, 12, 18 and 24 weeks). Significant differences were detected for the MFGM (mean 10·2 (95 % CI 8·33, 12·4) s) when compared with the placebo group (mean 7·61 (95 % CI 6·17, 9·30) s) (Fig. 2; *P* = 0·045)).

### Safety

In the present study, MFGM tablets were well tolerated by all participants who completed the 24-week trial. No subjective or objective adverse effects, including hepatic dysfunction and alterations in lipid metabolism, were observed, as shown in Table 4.

**Table 4.** Effect of milk fat globule membrane (MFGM) intervention on body weight, and clinical and biochemical parameters over time and compared with placebo (Mean values and standard deviations)

		Baseline†		12 weeks		24 weeks	
Parameter	Intervention*	Mean	sd	Mean	sd	Mean	sd
Body weight (kg)	MFGM	59·2	12·1	59·5	13·3	59·5	13·3
	Placebo	58·9	12·1	59·2	10·1	59·4	9·90
Leucocytes (×10^3^/μl)	MFGM	5·47	1·58	5·57	1·61	5·38	1·47
	Placebo	5·80	1·16	5·78	1·42	5·47	1·20
Erythrocytes (×10^6^/μl)	MFGM	4·50	0·38	4·52	0·39	4·52	0·34
	Placebo	4·53	0·40	4·45	0·38	4·49	0·38
Hb (g/l)	MFGM	138	11·3	138	11·6	138	10·6
	Placebo	140	12·6	138	11·7	138	12·1
Haematocrit (%)	MFGM	42·4	3·33	42·6	3·53	42·4	3·20
	Placebo	43·1	3·71	42·6	3·50	42·8	3·50
Glucose (mg/dl)‡	MFGM	98·8	20·2	94·9	15·2	97·0	19·0
	Placebo	98·4	27·8	94·6	16·1	95·6	15·6
HbA1c (%)	MFGM	5·86	0·48	5·88	0·53	5·82	0·50
	Placebo	5·95	0·83	5·88	0·44	5·92	0·51
Total cholesterol (mg/dl)‡	MFGM	209·9	36·2	211·6	36·6	215·1	39·4
	Placebo	207·5	40·2	211·3	38·9	216·1	39·5
LDL-cholesterol (mg/dl)‡	MFGM	119·7	32·8	119·4	32·0	121·9	33·2
	Placebo	120·8	36·8	119·9	35·1	125·3	37·3
HDL-cholesterol (mg/dl)‡	MFGM	67·4	15·9	67·9	16·6	67·8	18·5
	Placebo	64·6	16·8	67·1	18·5	67·2	17·9
TAG (mg/dl)‡	MFGM	109·9	102·3	106·5	60·9	115·5	84·3
	Placebo	116·5	88·9	111·8	103·8	110·9	70·4
ASAT (IU)	MFGM	26·6	16·1	24·9	11·3	24·8	10·6
	Placebo	23·4	9·20	25·0	10·0	24·9	9·90
ALAT (IU)	MFGM	23·9	16·3	22·8	15·0	22·1	13·9
	Placebo	22·9	15·1	25·1	18·9	23·3	15·7

ASAT, aspartate aminotransferase; ALAT, alanine aminotransferase.

* No significant differences were observed for any parameter between the interventions at each time point.

† No significant differences were observed during baseline, 12 weeks and 24 weeks between the interventions.

‡ To convert glucose from mg/dl to mmol/l, multiply by 0·0555; to convert cholesterol from mg/dl to mmol/l, multiply by 0·0259; to convert TAG from mg/dl to mmol/l, multiply by 0·0113.

## Discussion

Physical performance is fundamental for an active and independent life for all ages. The recent increase in the population of elderly people in developed countries has prompted the importance of physical activity to maintain healthy living. Physical performance relates not only to the musculoskeletal system, but also to other aspects of daily living. The U.S. Preventative Services Task Force Recommendation states that exercise or physical therapy and vitamin D supplementation can prevent falling in community-dwelling people aged 65 years or older, indicating the importance of nutritional supplementation^(^^24^^)^. Although many dietary supplements have been investigated for the prevention of household and recreational injuries, effective approaches remain to be elucidated especially for community-dwelling older adults and older adults in assisted living. With both populations were included inhabitants outside of either a nursing home or a hospital, a situation that contributed to the rapid growth of the population. Hence, due to this diversity among people, safe and handy dietary support is required to maintain physical performance^(^^25^^)^.

In the present study, supplementation with MFGM for 24 weeks did not lead to significant differences in isometric knee extension strength of each leg compared with that with placebo (the primary outcome of this study). The result of this study on leg extension contradicted the previous pilot trial with 10 weeks’ MFGM ingestion^(^^17^^)^. The most prominent differences between our study design and that of the previous study was the exclusion of additional mandatory exercise besides activities of daily life in this study^(^^17^^)^. All the studies conducted previously^(^^14^^–^^17^^)^ requested exercise ranging from 12 to 14 on the Borg Rate of Perceived Exertion scale in addition to normal physical activity. Kim *et al.*^(^^14^^)^ directly compared the effects of exercise and dietary MFGM in relation to frailty components, which included weight loss, exhaustion, low physical activity and slow walking. They reported that interventions, including exercise and nutrition such as MFGM, may improve frailty status. Other types of physical performance, including chair stand, chair stepping, and area of femoral muscle were similarly not changed in the present study, suggesting that additional exercise plus MFGM is an important component for these functions. Another difference of this study and the previous study is the timing for the intake of MFGM. The participants in the present study were asked to take MFGM or placebo after breakfast. In the previous study^(^^15^^)^, the subjects were instructed to consume the tablets within 1 h before the training on days with exercise training. These results suggested that MFGM may require additional exercise to improve physical performance, such as isometric extension strength of the upper leg musculature.

Interestingly, the time of the one-leg stance with eyes closed was found to be significantly increased in the MFGM group, although false-positive effects with potential selection bias could not be formally contradicted. The mixed-effect model for repeated measures under unstructured covariance structure revealed a significant difference between the MFGM and placebo groups. Interventional studies with MFGM, including the present study, have been performed over different periods ranging from 5 to 24 weeks, albeit using the same dose of MFGM (1 g MFGM per d throughout the studies^(^^14^^–^^17^^)^). Each study employed 1 g MFGM in six tablets per d for different intervention-periods, resulting in a total accumulation of MFGM of 35 g for 5 weeks^(^^16^^)^, 70 g for 10 weeks^(^^15^^,^^17^^)^, 84 g for 12 weeks^(^^14^^)^, and 98 g for 24 weeks (present study) in each trial. Differential effects on the physical performance of MFGM, such as agility, muscle mass and balance, may be focused on the existence of distinctive domains in physical performance and highlight the specificity of MFGM effectiveness with or without additional exercise.

There are several limitations to the present study. The most important limitation in the observations in this study derives from the failure of the primary outcome. Since all the components in the study were designed based on the primary outcome including sample number, the interpretation of secondary outcomes and generalisation for other populations should be carefully considered. The failure of a primary outcome significantly affects the statistical power for secondary outcomes. Pocock & Stone^(^^26^^)^ studied several clinical trials regarding the result of the primary outcome and the interpretation for those studies. They found significant misinterpretation of these studies including misjudging or overestimation of the outcomes. Based on this analysis, they indicated what to do next when a trial fails to produce a positive finding for its primary outcome. These include: (1) declare that the trial is positive due to the prespecified level; (2) improve the design of future trials; and (3) abandon the intervention as ineffective. Among these suggestions, the present study meets the situation for both (1) and (2), since this study has showed a possibly novel effect of MFGM on balance in a certain population. Moreover, this study focused on community-dwelling healthy participants ranging from 50 to 70 years old. An obvious limitation of the present observation is to generalise beyond the age including more elderly people. In addition, the study design did not specify the ratio between men and women; female participants were significantly dominant. Further studies should examine the relationship between MFGM and each physical function with different durations, dosage, sex and age. Cross-over studies maybe relevant to clarify the possible mechanisms between the exercise-dependent and exercise-independent effects of MFGM.

The effect of MFGM dose was also not studied in this intervention. Dose-dependent effects of MFGM may play a role in the result of the intervention. Unclear mechanisms underlying MFGM function are also an obvious and essential part of limitation for generalisation of the results with this study. Six tablets of MFGM contain apparently more protein (0·53 g) than those of placebo tablets (0·27 g); the difference could be significant for the physical performance of the participants (Table 1). We failed to detect statistically significant differences in any physical parameters including body weight (Table 2), isometric knee extension strength of each leg, the chair stand test, the chair stepping test, and the areas of VM. These parameters might be affected by the difference of protein composition in MFGM tablets, if any. Moreover, the mean daily protein intakes of Japanese adults indicated that Japanese adults of age 50–59 years consume a mean of 71·1 (sd 21·8) g and those aged 60–69 years consume 73·3 (sd 22·3) g^(^^27^^)^. The difference in protein composition between six tablets of MFGM and six tablets of placebo is 0·26 g per d, suggesting that the difference in protein intake is about 0·37–0·35 %. The placebo contains 1 g of milk powder which corresponds to about 20 ml of milk, whereas six tablets of MFGM corresponds to about 600 ml of milk^(^^15^^,^^28^^)^. Although placebo contains much less of milk derivatives compared with that of MFGM, it should incorporate a certain amount of MFGM. The possible effect of this MFGM in the placebo should be carefully considered and is an obvious point to be studied in a future trial. The typical composition of MFGM, including proteins and lipids, may play a role for the effectiveness of MFGM in physical performance. The identification of active composition is also an important aspect for further study. Furthermore, a follow-up study on the participants will give an additional insight of the effect of MFGM including the effective period on the time of one-leg stance with eyes closed. The interpretation and application of the results of this study should require consideration because of these selection bias.

In conclusion, the present study suggests that MFGM improved the physical performance related to balance in a community-dwelling cohort of Japanese adults in the absence of any mandatory exercise. Postural control for balance is a complex function involving several independent physiological systems in which vestibular function, visual function, muscle strength and sensory nervous system control balance, and its failure to do so has been closely correlated with falls^(^^29^^)^, indicating this as an important target for maintaining daily life activity in older adults. Several interventional studies have provided evidence that the approach for improving balance activity requires both exercise and nutrition^(^^3^^,^^24^^)^. These findings have an important implication to support safe and active living of older adults in living community and highlight the importance of dietary approaches relevant to the functional aspect of physical performance including neuromuscular junctions.

## References

[1] Vaiserman A & Lushchack O (2017) Implementation of longevity-promoting supplements and medications in public health practice: achievements, challenges and future perspectives. J Tranl Med 15, 160. 10.1186/s12967-017-1259-8 PMC5520340 28728596

[2] Chang CI, Huang KC, Chan DC, (2015) The impacts of sarcopenia and obesity on physical performance in the elderly. Obes Res Clin Pract 9, 256–265. 25175711 10.1016/j.orcp.2014.08.003

[3] Witard OC & Ball D (2018) The interaction between nutrition and exercise for promoting health and performance. Proc Nutr Soc 77, 1–3. 28721834 10.1017/S0029665117001100

[4] Sakellariou GK, Lightfoot AP, Earl KE, (2017) Redox homeostasis and age-related deficits in neuromuscular integrity and function. J Cachexia Sarcopenia Muscle 8, 881–906. 28744984 10.1002/jcsm.12223 PMC5700439

[5] Kwon YN & Yoon SS (2017) Sarcopenia: neurological point of view. J Bone Metab 24, 83–89. 28642851 10.11005/jbm.2017.24.2.83 PMC5472802

[6] Liu W, Klose A, Forman S, (2017) Loss of adult skeletal muscle stem cells drives age-related neuromuscular junction degeneration. eLife 6, e26464. 10.7554/eLife.26464 PMC5462534 28583253

[7] Inoue A, Cheng XW, Huang Z, (2017) Exercise restores muscle stem cell mobilization, regenerative capacity and muscle metabolic alterations via adiponectin/AdipoR1 activation in SAMP10 mice. J Cachexia Sarcopenia Muscle 8, 370–385. 27897419 10.1002/jcsm.12166 PMC5476856

[8] Tomàs J, Garcia N, Lanuza MA, (2017) Presynaptic membrane receptors modulate ACh release, axonal competition and synapse elimination during neuromuscular junction development. Front Mol Neurosci 10, 132. 28559796 10.3389/fnmol.2017.00132 PMC5432534

[9] Ishida Y, Kiyokawa Y, Asai T, (2016) Ameliorating effects of sphingomyelin-based liposomes on sarcopenia in senescence-accelerated mice. Biol Pharm Bull 39, 786–793. 27150148 10.1248/bpb.b15-00915

[10] Haramizu S, Ota N, Otsuka A, (2014) Dietary milk fat globule membrane improves endurance capacity in mice. Am J Physiol Regul Integr Comp Physiol 307, R1009–R1017. 25163913 10.1152/ajpregu.00004.2014

[11] Coto-Montes A, Boga JA, Tan DX, (2016) Melatonin as a potential agent in the treatment of sarcopenia. Int J Mol Sci 17, 1771. 10.3390/ijms17101771 PMC5085795 27783055

[12] Yano M, Minegishi Y, Sugita S, (2017) Milk fat globule membrane supplementation with voluntary running exercise attenuates age-related motor dysfunction by suppressing neuromuscular junction abnormalities in mice. Exp Gerontol 97, 29–37. 28729214 10.1016/j.exger.2017.07.012

[13] Haramizu S, Mori T, Yano M, (2014) Habitual exercise plus dietary supplementation with milk fat globule membrane improves muscle function deficits via neuromuscular development in senescence-accelerated mice. Springerplus 3, 339. 25110626 10.1186/2193-1801-3-339 PMC4125610

[14] Kim H, Suzuki T, Kim M, (2015) Effects of exercise and milk fat globule membrane (MFGM) supplementation on body composition, physical function, and hematological parameters in community-dwelling frail Japanese women: a randomized double blind, placebo-controlled, follow-up trial. PLOS ONE 10, e0116256. 10.1371/journal.pone.0116256 PMC4319727 25659147

[15] Ota N, Soga S, Hase T, (2015) Daily consumption of milk fat globule membrane plus habitual exercise improves physical performance in healthy middle-aged adults. Springerplus 4, 120. 10.1186/s40064-015-0896-8 PMC4369537 25810952

[16] Ota N, Soga S & Shimotoyodome A (2016) Dietary milk fat globule membrane with semiweekly light exercise improves choice stepping reaction time in healthy Japanese elderly subjects: a randomized double blind, placebo-controlled trial. J Ageing Res Clin Pract 5, 98–101.

[17] Minegishi Y, Ota N, Soga S, (2016) Effects of nutritional supplementation with milk fat globule membrane on physical and muscle function in healthy adults aged 60 and over with semiweekly light exercise: a randomized double-blind, placebo-controlled pilot trial. J Nutr Sci Vitaminol *(*Tokyo*)* 62, 409–415. 28202846 10.3177/jnsv.62.409

[18] Ross S (2016) Targeting the glycome of the milk fat globule membrane for antiinfective properties. PhD Thesis, National University of Ireland Galway.

[19] Cavaletto M, Giuffrida MG & Conti A (2008) Milk fat globule membrane components – a proteomic approach. Adv Exp Med Biol 606, 129–141. 18183927 10.1007/978-0-387-74087-4_4

[20] Drew MK & Finch CF (2016) The relationship between training load and injury, illness and soreness: a systematic and literature review. Sports Med 46, 861–883. 26822969 10.1007/s40279-015-0459-8

[21] Tate DF, Jackvony EH & Wing RR (2003) Effects of Internet behavioral counseling on weight loss in adults at risk for type 2 diabetes: a randomized trial. JAMA 289, 1833–1836. 12684363 10.1001/jama.289.14.1833

[22] Giorgetti MM, Harris BA & Jetta A (1998) Reliability of clinical outcome measures in the elderly. Physiother Res Int 3, 274–283. 9859135 10.1002/pri.150

[23] Lundin H, Saaf M, Strender LE, (2017) Gait speed and one-leg standing time each added to the predictive ability of FRAX. Osteoporos Int 28, 179–187. 27844133 10.1007/s00198-016-3818-x PMC5206249

[24] Moyer VA, U.S. Preventive Services Task Force (2012) Prevention of falls in community-dwelling older adults: U.S. Preventive Services Task Force recommendation statement. Ann Intern Med 157, 197–204. 22868837 10.7326/0003-4819-157-3-201208070-00462

[25] Alvarez KJ, Kirchner S, Chu S, (2015) Falls reduction and exercise training in an assisted living population. J Aging Res 2015, 957598. 10.1155/2015/957598 PMC4541005 26345431

[26] Pocock SJ & Stone GW (2016) The primary outcome fails – what next? NEJM 375, 861–870. 27579636 10.1056/NEJMra1510064

[27] Ministry of Health, Labour and Welfare (2018) Protein intake. Annual trend of average value and standard deviation of protein intake (by sex and age group). http://www.mhlw.go.jp/seisakunitsuite/bunya/kenkou_iryou/kenkou/kenkounippon21/eiyouchousa/keinen_henka_eiyou.html (accessed February 2018).

[28] Vesper H, Schmelz EM, Nikolova-Karashian MN, (1999) Sphingolipids in food and the emerging importance of sphingolipids to nutrition. J Nutr 129, 1239–1250. 10395583 10.1093/jn/129.7.1239

[29] Fernando E, Fraser M, Hendriksen J, (2017) Risk factors associated with falls in older adults with dementia: a systematic review. Physiother Can 69, 161–170. 28539696 10.3138/ptc.2016-14 PMC5435396

